# An investigation into corneal enzymatic resistance following epithelium-off and epithelium-on corneal cross-linking protocols

**DOI:** 10.1016/j.exer.2016.10.014

**Published:** 2016-12

**Authors:** Nada H. Aldahlawi, Sally Hayes, David P.S. O'Brart, Naomi D. O'Brart, Keith M. Meek

**Affiliations:** a Structural Biophysics Research Group, School of Optometry and Vision Sciences, Cardiff University, Maindy Road, Cardiff, CF24 4HQ, UK; b Keratoconus Research Institute, Department of Ophthalmology, St Thomas Hospital, London, SE1 7EH, UK

**Keywords:** Keratoconus, Cornea, Cross-linking, Epithelium-off, Epithelium-on, CXL, Enzymatic digestion

## Abstract

The aim of this study was to investigate corneal enzymatic resistance following epithelium off and on riboflavin/UVA cross-linking (CXL). One hundred and fourteen porcine eyes were divided into four non-irradiated control groups and seven CXL groups. The latter comprised; (i) epithelium-off, 0.1% iso-osmolar riboflavin, 9 mW UVA irradiation for 10 min, (ii) disrupted epithelium, 0.1% hypo-osmolar riboflavin, 9 mW UVA for 10 min, (iii) epithelium-on, 0.25% hypo-osmolar riboflavin with 0.01% benzylalkonium chloride (BACS), 9 mW UVA for 10 min, (iv) epithelium-on, 5 min iontophoresis at 0.1 mA for 5 min with 0.1% riboflavin solution, 9 mW UVA for 10 min or (v) 12.5 min, (vi) epithelium-on, prolonged iontophoresis protocol of 25 min with 1.0 mA for 5 min and 0.5 mA for 5 min with 0.25% riboflavin with 0.01% BACS, 9 mW UVA for 10 min or (vii) 12.5 min. Enzymatic resistance was assessed by daily measurement of a corneal button placed in pepsin solution and measurement of corneal button dry weight after 11 days of digestion. This study revealed that the enzymatic resistance was greater in CXL corneas than non-irradiated corneas (p < 0.0001). Epithelium-off CXL showed the greatest enzymatic resistance (p < 0.0001). The prolonged iontophoresis protocol was found to be superior to all other trans-epithelial protocols (p < 0.0001). A 25% increase in UVA radiance significantly increased corneal enzymatic resistance (p < 0.0001). In conclusion, although epithelium-on CXL appears to be inferior to epithelium-off CXL in terms of enzymatic resistance to pepsin digestion, the outcome of epithelium-on CXL may be significantly improved through the use of higher concentrations of riboflavin solution, a longer duration of iontophoresis and an increase in UVA radiance.

## Highlights

• Epi-off CXL is superior to epi-on CXL in terms of enzymatic resistance.
• Epi-on CXL effectiveness can be improved by using a prolonged iontophoresis protocol.
• Higher riboflavin concentrations and increased UVA radiance may also enhance epi-on CXL.

## 1 Introduction

Over the past decade, riboflavin/UVA corneal cross-linking (CXL) has become an established treatment to halt the progression of keratoconus and other corneal ectasias. Riboflavin is a hydrophilic molecule, with a molecular weight of 340 Da, while, the corneal epithelium is lipophyllic, with a decreasing permeability to molecules over 180 Da (Huang et al., 1989). Therefore in the “gold-standard” CXL protocol the epithelium is removed from the central corneal to allow adequate stromal absorption of riboflavin prior to UVA irradiation. Spoerl et al. confirmed this need for epithelial removal, reporting no changes in corneal biomechanics when CXL was performed with the epithelium intact (Spoerl et al., 1998). On this basis, the epithelium was removed in the first published clinical studies (Caporossi et al., 2006, Gkika et al., 2011, O'Brart et al., 2011, Richoz et al., 2013, Wittig-Silva et al., 2014, Wollensak et al., 2003). However, epithelial debridement is associated with a number of adverse events, including severe ocular pain in the immediate post-operative period, delayed visual rehabilitation and the risks of scarring, infectious and non-infectious keratitis (Koller et al., 2009).

As a result of such considerations, a number of trans-epithelial CXL techniques have been postulated. The first epithelium-on CXL (epi-on-CXL) protocols included the use of multiple topical applications of tetracaine 1% to disrupt epithelial tight junctions (Boxer Wachler et al., 2010, Chan et al., 2007) and partial epithelial debridement in a grid pattern (Rechichi et al., 2013). More recently, novel formulations of riboflavin have been developed to facilitate epi-on-CXL. Laboratory studies have shown that riboflavin preparations in which trometamol (Tris-hydroxymethyl-aminometane) and sodium ethylenediaminetetraacetic acid (EDTA) have been added facilitate stromal absorption when used in conjunction with superficial/grid pattern epithelial trauma (Alhamad et al., 2012). Similarly, preparations without dextran but with sodium chloride 0.44% and benzalkonium chloride (BAC) have been shown to facilitate trans-epithelial riboflavin stromal absorption (Kissner et al., 2010, Raiskup et al., 2012). Clinical studies with such formulations have demonstrated equivocal results with some suggesting similar efficacy to epithelium-off CXL (epi-off-CXL) (Filippello et al., 2012, Magli et al., 2013), and others showing less pronounced effects (Buzzonetti and Petrocelli, 2012, Kocak et al., 2014, Koppen et al., 2012, Leccisotti and Islam, 2010). As riboflavin is negatively charged at physiological pH and soluble in water, iontophoresis as a means of enhancing trans-epithelial absorption has also been postulated. In vitro studies of CXL using iontophoresis-assisted delivery (Ion-CXL) of riboflavin 0.1% with a current of 0.5–1 mA for 5–10 min have been encouraging, demonstrating enhanced trans-epithelial riboflavin absorption and corneal tissue biomechanics (Cassagne et al., 2016, Lombardo et al., 2014, Mastropasqua et al., 2014, Touboul et al., 2014, Vinciguerra et al., 2014a). Early clinical studies have reported cessation of progression and improvements in keratometric and visual parameters (Bikbova and Bikbov, 2014, Buzzonetti et al., 2015, Vinciguerra et al., 2014b).

In addition to the desire to keep the epithelium intact, the current “gold-standard”, epi-off-CXL protocol, involves a 30 min application of riboflavin followed by a 30 min irradiation with 370 nm UVA light, with an intensity of 3 mW/cm^2^, necessitating in excess of one hour treatment time. In an attempt to reduce treatment times, the use of accelerated CXL (A-CXL) procedures using the same energy dose but higher UVA intensities and shorter exposure times have been investigated. Published clinical studies of A-CXL protocols are relatively few. However, those using 7 mW for 15 min or 9 mW for 10 min have reported improvements in corrected distance acuity and a reduction in topographic keratometry at up to 46 months follow-up with no adverse events associated with the high fluences used (Cinar et al., 2014, Cummings et al., 2016, Kanellopoulos, 2012, Kymionis et al., 2014a, Shetty et al., 2015). More recently, it has also been demonstrated that the efficacy of A-CXL may be further improved by increasing the UVA exposure time and the overall cumulative dosage (Aldahlawi et al., 2016, Kymionis et al., 2014b, Sherif, 2014).

Increased resistance of the corneal stroma to enzymatic digestion following standard (Hayes et al., 2013, Spoerl et al., 2004) and accelerated (Aldahlawi et al., 2015) epi-off-CXL has been demonstrated by a number of investigators. It is likely that the improved enzymatic resistance is an important factor in protection against disease progression, since an increase in proteinase activity and a reduction in proteinase inhibitor activity has been identified in keratoconic corneas (Zhou et al., 1998). In order to investigate the efficacy of a number of different epi-on-CXL protocols, we compared the enzymatic resistance of corneal tissue cross-linked using epi-off-CXL with that of corneas treated with partially disrupted-epithelium CXL (dis-CXL), existing epi-on-CXL protocols (involving different riboflavin formulations and modes of delivery) and a prolonged iontophoresis CXL protocol with 0.25% riboflavin (TC-ion-CXL) that we have recently developed. In addition to this we examined the effect of increasing the cumulative UVA dosage on the enzymatic resistance of corneas treated with iontophoresis-assisted epi-on-CXL. Pepsin was selected as the enzyme of choice for this study in favour of collagenase, as it is a non-specific endopeptidase that can break down both collagen and proteoglycan core proteins, both of which are believed to be sites of riboflavin/UVA induced cross-links (Hayes et al., 2013). Porcine eyes were used as, unlike human cadaver eyes and rabbit eyes which are of limited availability in the UK, they were readily available in the fresh state and in the large numbers required for this study. However, due to the porcine cornea having a thicker epithelium than the human cornea, the results presented herein should be regarded as a conservative assessment of the effectiveness of trans-epithelial CXL.

## 2 Materials and methods

### 2.1 Specimen preparation

A total of one hundred and fourteen fresh porcine cadaver eyes with clear corneas and intact epithelium were retrieved from a local European Community licensed abattoir within 6–8 h of death. Due to the large number of eyes and treatment groups involved, it was necessary to split the study into two runs. Run 1 examined the effectiveness of dis-CXL and epi-on-CXL protocols at increasing corneal enzymatic resistance to digestion with pepsin, whilst run 2 examined the effect on enzymatic resistance of increasing the UVA dosage by 25% from 5.4 J/cm^2^ to 6.75 J/cm^2^ during iontophoretic epi-on-CXL. In both runs, the effectiveness of the standard epi-off CXL protocol at increasing enzymatic resistance was also examined for comparative purposes. The 11 treatment groups are described below and summarised in Table 1.

**Run 1:**

Each treatment group consisted of six eyes.

**i) Epi-off standard protocol (Group 1: Epi-off-ribo; Group 2: Epi-off-CXL)**

Complete corneal epithelial debridement was performed in groups 1 and 2 using a single edged razor blade. These eyes then received 0.1% riboflavin eye drops containing 20% dextran T-500 solution (Mediocross D^®^, Peschke Meditrade, Huenenberg, Switzerland) every 5 min for 30 min. The central 9 mm region of corneas in group 2 was then exposed to 365 nm UVA light with a fluence of CXL 9 mW/cm^2^ for 10 min using a CCL-365 Vario™ cross-linking system (Peschkmed, Huenenberg, Switzerland). During irradiation, riboflavin was re-applied at 5 min intervals. Group 1 served as a non-irradiated control.

**ii) Epi-disrupted protocol (Group 3: Dis-ribo; Group 4: Dis-CXL)**

Partial epithelial disruption was performed in groups 3 and 4, by making 64 full-thickness epithelial punctures with a 25 gauge needle in an 8 × 8 grid pattern. The corneas were then soaked in riboflavin 0.1% dextran-free solution (Vitamin B2 Streuli, Uznach, Switzerland) for 30 min and rinsed with phosphate-buffered saline (PBS) for 5 min. Group 4 was then irradiated with 9 mW UVA for 10 min with PBS applied at 5 min intervals to keep the corneal surface moist. Group 3 served as a non-irradiated control.

**iii) Epi-on and high riboflavin concentration protocol (Group 5: Medio-ribo; Group 6: Medio-CXL)**

Groups 5 and 6 received 0.25% riboflavin with 1.2% hydroxypropylmethyl cellulose (HPMC) and 0.01% BAC (Mediocross TE^®^, Peschketrade, Huenenberg, Switzerland) every 5 min for 30 min. This was followed by a 5 min rinse with PBS. Group 6 was then irradiated with 9 mW UVA for 10 min with PBS applied at 5 min intervals. Group 5 served as a non-irradiated control.

**iv) Epi-on, high riboflavin concentration and prolonged iontophoresis (St. Thomas-Cardiff) protocol (Group 7: TC-ion-ribo; Group 8: TC-ion-CXL)**

**Fig. 1:**
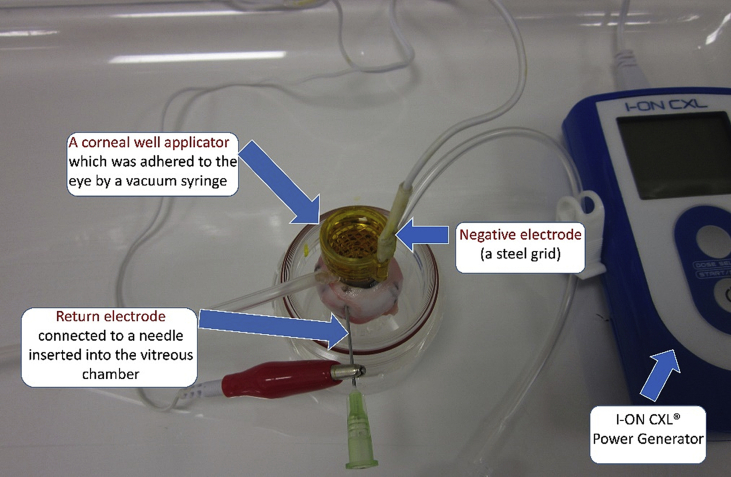
Iontophoresis riboflavin delivery system modified for use in ex-vivo eyes.

Groups 7 and 8 received iontophoresis assisted delivery of 0.25% riboflavin with 1.2% HPMC and 0.01% BAC (Mediocross TE^®^, Peschketrade, Huenenberg, Switzerland) using a current of 1 mA for 5 min. The corneas were then soaked with this riboflavin solution for 5 min before a further application of iontophoresis with a power of 0.5 mA/min for 5 min and another 5 min riboflavin soak. The iontophoresis delivery system was used to treat ex vivo eyes by connecting the return electrode to a needle inserted into the vitreous chamber; the negative electrode was a steel grid contained in a corneal well applicator which was adhered to the eye by means of a vacuum well system (Fig. 1). The steel grid (negative electrode) was completely covered with riboflavin and the power generator set to the desired current and duration. The steel grid remained covered with riboflavin solution for the entire procedure. After treatment the applicator was removed from the cornea and the corneas were washed with PBS for 5 min. Group 8 was then irradiated with 9 mW UVA for 10min with PBS applied at 5 min intervals. Group 7 served as a non-irradiated control.

**Run 2:**

Each treatment group consisted of 11 eyes.

**i) Epi-off standard protocol (Group 1: Epi-off-ribo; Group 2: Epi-off-CXL 5.4 J/cm**^2^**)**

Corneas were treated as described for groups 1 and 2 in run 1.

**ii) Epi-on, high riboflavin concentration and prolonged iontophoresis (St. Thomas-Cardiff) protocol (Group 8: TC-ion-CXL 5.4 J/cm**^2^**; Group 9: TC-ion-CXL 6.75 J/cm**^2^**)**

Groups 8 and 9 received iontophoresis assisted delivery of 0.25% riboflavin with 1.2% HPMC and 0.01% BAC (Mediocross TE^®^, Peschketrade, Huenenberg, Switzerland) using a current of 1 mA for 5min. The corneas were then soaked with this riboflavin solution for 5 min before a further application of iontophoresis with a power of 0.5 mA/min for 5 min and another 5 min riboflavin soak. Following a 3 min wash with PBS, the corneas in Group 8 were irradiated with 9 mW UVA for 10 min and those in Group 9 were irradiated with 9 mW UVA for 12min and 30 s. During irradiation, PBS was applied to all corneas at 5 min intervals.

**iii) Epi-on basic iontophoresis protocol (Group 10: Ion-CXL 5.4 J/cm**^2^**; Group 11: Ion-CXL 6.75 J/cm**^2^**)**

Groups 10 and 11 underwent iontophoresis assisted riboflavin delivery using an isotonic 0.1% riboflavin solution (Vitamin B2) containing 1.0% HPMC dextran-free solution (Mediocross M^®^, Peschketrade, Huenenberg, Switzerland) and a 1 mA current (Iontophoresis device, Sooft Italia S.p.A, Italy). After treatment the corneas were washed with PBS for 3 min. Group 10 was then irradiated with 9 mW UVA for 10min and group 11 was irradiated with 9 mW UVA for 12 min and 30 s. During irradiation, PBS was applied to all corneas at 5 min intervals.

### 2.2 Measurements of corneal thickness

Using a Pachette2™ Ultrasonic Pachymeter (DGH Technology, Exton, USA), the central corneal thickness was measured in all eyes prior to treatment and where applicable after removal of the epithelium, application of riboflavin and UVA irradiation.

### 2.3 Measurements of enzymatic digestion

A corneo-scleral ring was dissected from each eye immediately following treatment, wrapped tightly in Clingfilm™ (to prevent moisture loss) and refrigerated until all treatments were complete. An 8 mm corneal button was trephined from the centre of each cornea using a disposable skin biopsy punch. The corneal buttons were then immersed into individual plastic tubes, each containing 5 ml of pepsin solution, and incubated in a water bath at a temperature of 23 °C. The pepsin solution was made up of 1 g of >500 U/mg pepsin from porcine gastric mucosa (Sigma-Aldrich, Dorset, UK) in 10 ml 0.1 M HCL at pH 1.2. The structural integrity of the most anterior layers of the cornea was assessed by means of daily measurements of corneal button diameter. The measurements, which were made using an electronic digital caliper, continued until the specimen could no longer be distinguished from the surrounding pepsin solution. At this point the tissue was considered to have undergone complete digestion.

As an additional means of assessing enzymatic resistance, 5 corneal buttons from each of the 6 treatment groups in run 2 were removed after 11 days in pepsin digest solution and placed in a 60 °C oven until a constant dry weight was obtained. The dry weight of the tissue represents the total mass of undigested tissue and can therefore be used as an indicator of the effective depth of cross-linking.

### 2.4 Statistical evaluation

Measurements of corneal thickness, complete digestion time and tissue dry weight were statistically analysed using a one-way analysis of variance (ANOVA) test. Post hoc Bonferroni comparisons were used to isolate significant interactions. All statistical analyses were performed with the Statistical Package for the Social Sciences (IBM SPSS Statistics 20, New York, USA). P < 0.01 was considered significant. Data were presented in the results as mean ± standard deviation (SD).

## 3 Results

### 3.1 Corneal thickness

**Fig. 2:**
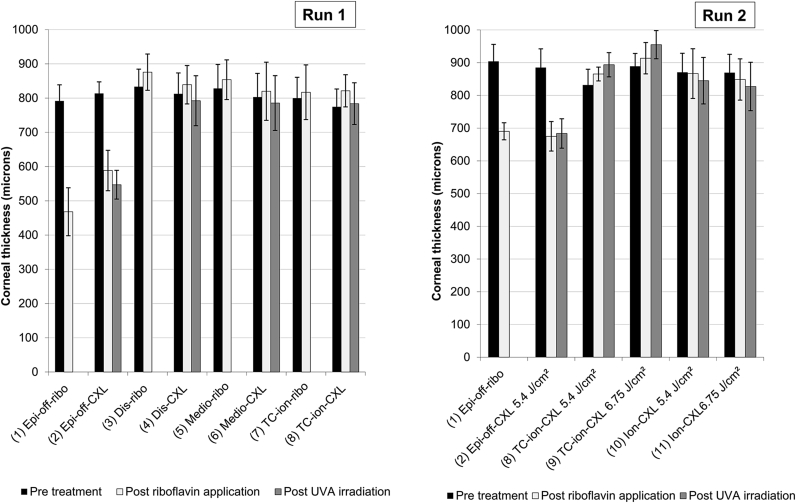
Corneal thickness measurements are shown for each group in run 1 and run 2 before treatment, after riboflavin application and where applicable, following UVA irradiation. *In groups 1 and 2 the corneal epithelium (measuring ∼90 μm in thickness) was removed as part of the riboflavin application process.

Fig. 2 shows the average corneal thickness pre-treatment, post-riboflavin application and post-irradiation for each treatment group in runs 1 and 2. Statistical tests revealed that corneas treated using the standard, epi-off protocol and an iso-osmolar riboflavin solution (groups 1 and 2) were significantly thinner than the corneas in all other groups (p < 0.007).

### 3.2 Qualitative assessment of riboflavin uptake

**Fig. 3:**
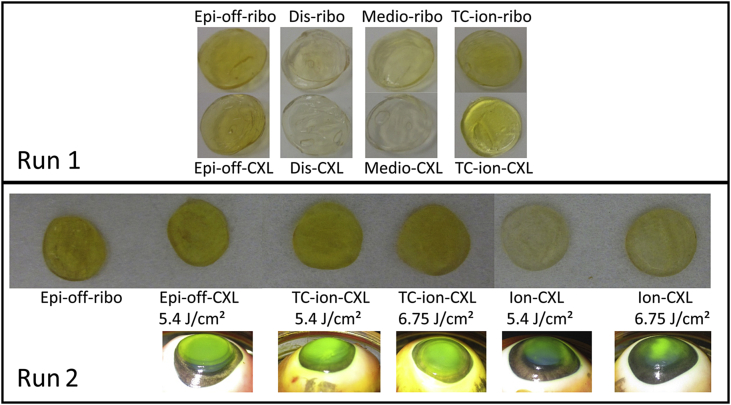
Corneal buttons from each group in run 1 and 2 are shown immediately post-treatment. The characteristic yellow colour of riboflavin can be seen most clearly in the epithelium-removed, riboflavin treated corneas (epi-off) and in the corneas that received riboflavin via the St Thomas's/Cardiff modified iontophoresis protocol (TC-Ion). Photographs recorded during the irradiation process show a non-homogenous distribution of riboflavin in corneas treated with the basic iontophoresis protocol (Ion-CXL).

As shown in Fig. 3, the distinctive yellow colouration of riboflavin was most clearly visible in the epi-off and TC-ion treated corneas. Although riboflavin was also seen in corneas from other treatment groups, the colour was notably less intense. Photographs recorded during the irradiation process in run 2 showed a non-homogenous distribution of riboflavin in Ion-CXL treated corneas and a more uniform distribution following epi-off CXL and TC-Ion-CXL.

### 3.3 Pepsin digestion of corneal buttons

**Fig. 4:**
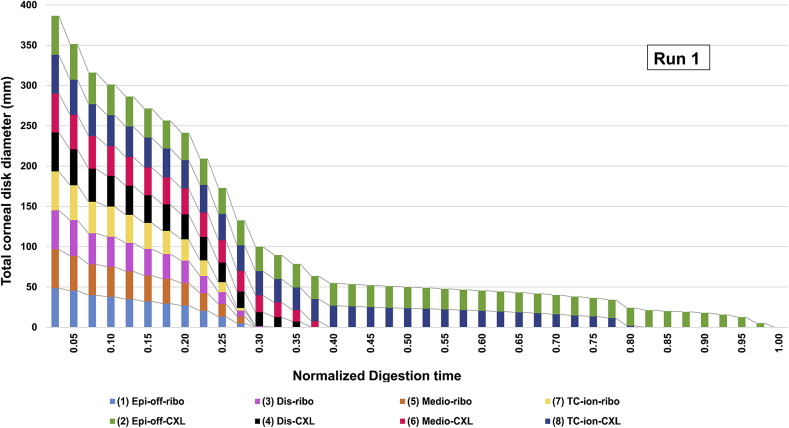
The summed diameter of all corneal disks within each treatment group (n = 6) are shown for run 1 as a function of time in pepsin digest solution. The digestion time for each treatment group has been normalized against the total digestion time of the standard epi-off CXL group.

**Fig. 5:**
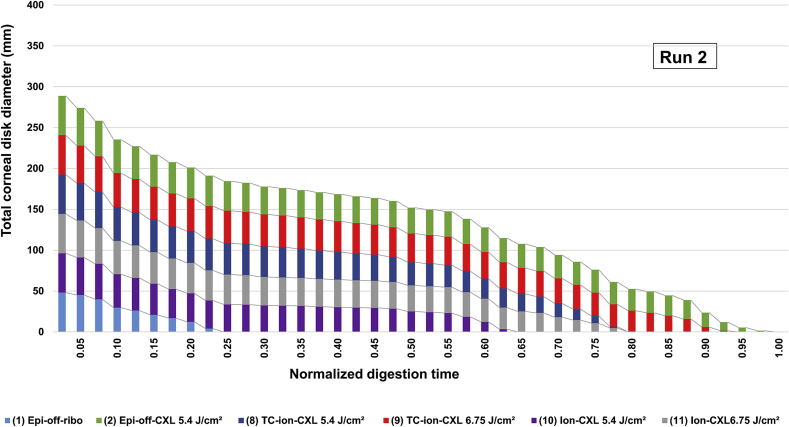
The summed diameter of all corneal disks within each treatment group (n = 6) are shown for run 2 as a function of time in pepsin digest solution. The digestion time for each treatment group has been normalized against the total digestion time of the standard epi-off CXL group.

Table 2 shows the number of days required for complete tissue digestion to occur in each irradiated and non-irradiated treatment group. Although the digestion times of equivalent non-irradiated (group 1) and cross-linked treatment groups (group 2) were seen to vary slightly between runs 1 and 2, possibly as a result of differences in the breed and age of the pig eyes, the overall trends were consistent between the two runs. For this reason the data from each run was normalized against the total digestion time of the standard epi-off CXL group to facilitate comparison between the two runs (Fig. 4, Fig. 5).

Fig. 4, Fig. 5 show cumulative measurements of corneal disk diameter for each irradiated and non-irradiated treatment group throughout the digestion process. In both runs 1 and 2, the cross-linked groups showed a significantly greater resistance to enzymatic digestion than the non-irradiated groups (p < 0.0001). In run 1, complete digestion of all non-irradiated corneas had occurred by day 13 (Table 2). At the same time point (normalized digestion time of 0.33), the mean diameter of the epi-off-CXL, dis-CXL, medio-CXL and TC-ion-CXL groups had only decreased by 39%, 62%, 74%, and 40% respectively (Fig. 4). No significant difference was found between the non-irradiated groups in terms of either the average corneal button diameter at any time point in the digestion process or in the time required for complete digestion to occur (Fig. 4). There were however significant differences between the cross-linked groups in term of the time taken for complete digestion to occur (Fig. 4). The conventionally treated, epi-off-CXL corneas (group 2) took significantly longer to digest than all other cross-linked corneas (p < 0.0001). Although less resistant to enzyme digestion than the epi-off-CXL corneas, the TC-ion-CXL treated corneas (group 8) took significantly longer to digest than corneas treated with other disrupted epithelium (dis-CXL) or epi-on-CXL protocols (p < 0.0001).

In run 2, the non-irradiated corneas were completely digested by day 10 (Table 2). At this same time point, which corresponds to a normalized digestion time of 0.25, the mean diameter of corneas in the epi-off-CXL 5.4 J/cm^2^, TC-ion-CXL 5.4 J/cm^2^, TC-ion-CXL 6.75 J/cm^2^, Ion-CXL 5.4 J/cm^2^ and Ion-CXL 6.75 J/cm^2^ treatment groups had reduced by 21.4%, 16.2%, 13.4%, 26.7% and 19.4% respectively (Fig. 5). Significant differences in the mean disk diameter of the CXL treatment groups were only apparent after 24 days of digestion (corresponding to a normalized digestion time of 0.6 in Fig. 5). The epi-off-CXL group (group 2) took longer to undergo complete digestion than all other cross-linked groups (p < 0.0001) but corneas treated with the prolonged, high riboflavin concentration, iontophoresis protocol (TC-ion-CXL) were found to persist in the pepsin digest solution for significantly longer than those treated with the basic Ion-CXL protocol (Fig. 5). In both the Ion-CXL and TC-ion-CXL treatment groups, an increase in UVA radiance from 5.4 J/cm^2^ to 6.75 J/cm^2^ resulted in a significant increase in the time required for complete digestion to occur (P < 0.0001).

### 3.4 Undigested tissue mass

**Fig. 6:**
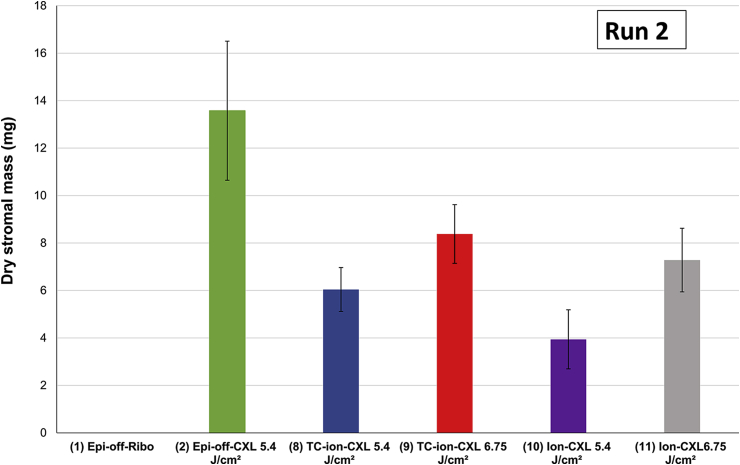
Corneal button dry weight after 11 days of digestion. Error bars show standard deviation.

In run 2, only the cross-linked corneas remained after 11 days in pepsin digest solution (Fig. 6). At this time point, the average stromal dry weight of the epi-off-CXL 5.4 J/cm^2^ treated corneas (group 2) was significantly higher than that of all other treatment groups (groups 8, 10, 11, P < 0.0001; group 9, P < 0.001). The stromal dry weight did not differ significantly between the Ion-CXL 5.4 J/cm^2^ and Ion-CXL 6.75 J/cm^2^ treatment groups (p = 0.32) or between the TC-ion-CXL 5.4 J/cm^2^ and TC-ion-CXL 6.75 J/cm^2^ groups (p = 0.038). However, corneas treated with the TC-Ion-CXL 6.75 J/cm^2^ protocol had a higher stromal dry weight than corneas treated with basic ion-CXL 5.4 J/cm^2^ protocol (p < 0.003).

## 4 Discussion

Attaining adequate stromal riboflavin concentration is essential for CXL. Riboflavin acts as a photo-sensitizer for the production of oxygen free radicals, which drive the CXL process (McCall et al., 2010) and as a result insufficient stromal absorption may result in treatment failure (Wollensak et al., 2003, Wollensak and Iomdina, 2009). As riboflavin is a hydrophilic molecule which cannot penetrate epithelial cell membranes and tight junctions, the first riboflavin/UVA CXL protocols required complete central corneal epithelial debridement prior to riboflavin application (Spoerl et al., 1998, Wollensak et al., 2003). Although epi-off-CXL is considered to be the gold standard procedure, with multiple prospective and randomized controlled studies demonstrating its efficacy in terms of cessation of keratoconus progression and improvements in keratometric and visual parameters (Caporossi et al., 2006, Gkika et al., 2011, O'Brart et al., 2011, Richoz et al., 2013, Wittig-Silva et al., 2014, Wollensak et al., 2003), new methods of epi-on-CXL have been postulated to remove the need for epithelial debridement, thereby alleviating post-operative pain and potential risks of infection (Koller et al., 2009). In addition, to reduce the long treatment times associated with epi-off-CXL with 3mw/cm^2^ UVA irradiation, the use of A-CXL protocols using the same energy dose but higher UVA intensities and shorter exposure times have been advocated. In order to evaluate the efficacy of such differing CXL protocols, multiple methodologies, including extensiometry (Cassagne et al., 2016, Kissner et al., 2010, Vinciguerra et al., 2014a, Wollensak and Iomdina, 2009), shear wave elastography (Touboul et al., 2014), Brillouin microscopy (Scarcelli et al., 2013) and Scheimpflug air pulse tonometry (Mastropasqua et al., 2014) have been employed. However each of these methodologies have their inherent inaccuracies and as yet there is no agreed best practice for assessing biomechanical changes following CXL.

Although the precise aetiology of keratoconus is unknown (Davidson et al., 2014), an increased activity of protease enzymes and reduced activity of protease inhibitors has been identified (Spoerl et al., 2004), with the resultant increase in stromal protein digestion liable to be a factor in corneal thinning and secondary biomechanical instability (Andreassen et al., 1980). Spoerl et al. (2004) reported increased resistance of stromal tissue to enzymatic digestion following epi-off CXL with a dose response related to the intensity of UVA irradiance. This increased resistance to protease digestion following CXL has been replicated by others (Hayes et al., 2013, Aldahlawi et al., 2016) and is thought to be an important factor in preventing disease progression. In this study we utilized enzymatic resistance to pepsin digestion to evaluate the effects of epi-off-CXL, dis-CXL and epi-on-CXL. Although minor differences in the rate of enzymatic digestion were seen between similarly treated corneas in runs 1 and 2, possibly due to variations in the age and breed of the pig eyes in different batches of abattoir tissue, each run showed an increased resistance in all of the cross-linked groups compared to their non-irradiated controls. However, in accordance with the findings of Wollensak and Iomdina (2009), which reported corneal stiffness after epi-on-CXL to be only one fifth of that seen after epi-off-CXL (indicating a reduced crosslinking effect), we found enzymatic resistance to be significantly greater with epi-off-CXL than with dis-CXL or epi-on-CXL. Whilst dis-CXL showed a greater resistance to digestion than the non-irradiated controls, it was not as effective as the other epi-on-CXL protocols tested, possibly due to the non-homogeneous uptake of riboflavin that we have documented previously (Alhamad et al., 2012). Such observations are supported by a recently published, randomized controlled study which showed better corneal flattening with epi-off-CXL than dis-CXL, although interestingly, dis-CXL resulted in better improvement of corrected distance visual acuity at 6 months (Razmjoo et al., 2014).

In this study we showed that corneas cross-linked using Mediocross TE^®^ (Medio-CXL protocol) showed a greater resistance to enzymatic digestion than non-irradiated controls and dis-CXL treated corneas. However, the enhanced enzymatic resistance achieved with Medio-CXL was found to be inferior to that of the epi-off and ion-CXL protocols. Mediocross TE^®^ is a hypo-osmolar riboflavin solution that contains 0.01% BAC, a cationic surfactant that can disrupt epithelial tight junctions to increase corneal permeability to riboflavin (Kissner et al., 2010, Raiskup et al., 2012). Laboratory studies have shown that preparations with sodium chloride and BAC can facilitate riboflavin transfer through an intact epithelium albeit in a reduced concentration compared to the epi-off-CXL (Touboul et al., 2014), and with significant associated superficial epithelial damage (Uematsu et al., 2007). Published clinical studies using enhanced riboflavin solutions with epithelial penetration enhancers are limited and have produced equivocal results. Some have reported similar efficacy to epi-off-CXL (Filippello et al., 2012, Magli et al., 2013), while others have demonstrated less pronounced effects with high rates of treatment failure (Buzzonetti and Petrocelli, 2012, Kocak et al., 2014, Koppen et al., 2012, Leccisotti and Islam, 2010). There are currently only three published randomized, controlled trials comparing epi-off and epi-on-CXL. Two of the studies, with follow-up times of up to 12 months, reported similar outcomes (Nawaz et al., 2015, Rossi et al., 2015). However, the third study, showed epi-on-CXL to be safe but demonstrated continued progression of keratoconus in 23% of cases at 12 month follow-up (Soeters et al., 2015). The latter result is consistent with the less pronounced biomechanical changes observed experimentally following epi-on-CXL (Wollensak and Iomdina, 2009).

As riboflavin is negatively charged at physiological pH and soluble in water, the use of iontophoresis has been postulated to enhance trans-epithelial absorption. Commercially recommended protocols for iontophoresis utilize 0.1% riboflavin solution and electrical currents of 0.5–1 mA for 5–10 min. Treatment of rabbit corneas by this means has been shown to reduce the stromal riboflavin uptake achieved with epi-off-CXL by two thirds, but to produce a similar improvement in corneal biomechanics (Cassagne et al., 2016). Studies on rabbit and human cadaver corneas have demonstrated similar findings, with better riboflavin penetration and increased elastometry measurements obtained with Ion-CXL than with epi-on-CXL (using Ricrolin TE) but less than epi-off-CXL (Vinciguerra et al., 2014a). In a human donor eye model, Mastropasqua et al. demonstrated a corneal stiffening effect following Ion-CXL (Mastropasqua et al., 2014), with Lombardo et al. (2014) reporting an almost comparable effect on corneal stiffness to that with epi-off-CXL. These findings have been confirmed by supersonic shear wave elastography (Touboul et al., 2014). Furthermore, initial clinical studies in prospective case series have reported cessation of keratoconus progression with up to 15 month follow-up and some limited improvements in keratometric and visual parameters (Bikbova and Bikbov, 2014, Buzzonetti et al., 2015, Vinciguerra et al., 2014b). However, the relative efficacy of this technique compared to epi-off-CXL remains to be determined especially over longer term follow-up.

Using spectrophotometry (Hayes et al., 2015) and two-photon fluorescence microscopy (Gore et al., 2014), we have found that stromal riboflavin absorption in epithelium-intact corneas can be improved by increasing riboflavin concentration, epithelial contact time and iontophoresis dosage. Based on this work, we have modified the basic Sooft italia Ion-CXL protocol (Sooft italia S.p.A, Motegiorgio, Italy) and developed the St Thomas's/Cardiff modified iontophoresis protocol (TC-ion-CXL) which uses Mediocross TE^®^ as the iontophoretic solution instead of Ricrolin+^®^, as the riboflavin concentration of the former is higher (0.25%) and the use of the cationic surfactant BAC has been shown with percutaneous treatment to have synergistic effect with iontophoresis on the transport of anions (Fang et al., 1998). In addition to using this formulation, the modified protocol employed a riboflavin-epithelial contact period of 5 min after iontophoresis to allow time for the sub-epithelial iontophoretically delivered riboflavin to diffuse homogeneously into the stroma, as well as an increased dosage of iontophoresis with a second treatment 5 min after the first. Results from the present study demonstrated that both the basic Ion-CXL and modified TC-ion-CXL protocols resulted in a greater resistance to enzymatic digestion than all other dis-CXL and trans-epithelial CXL protocols tested. Furthermore, as we have demonstrated previously for epi-off CXL treated corneas (Aldahlawi et al., 2016), we have now shown that the enzymatic resistance of Ion-CXL and TC-ion-CXL treated corneas can also be enhanced by increasing the cumulative energy dose and allowing additional type I photochemical cross-linking to occur. The improvement in enzymatic resistance was evidenced by the Ion-CXL and TC-ion-CXL corneas treated with a total energy dose of 6.75 J/cm^2^ persisting for longer in enzyme digest solution than those that received a lower energy dose of 5.4 J/cm^2^. The absence of any difference in their dry weights after 11 days of digestion is likely explained by the fact that at this stage of the digestion process, the rate of digestion was similar for both the high and low energy treatment groups and significant differences in average corneal disk diameter were not observed until day 24.

The enzymatic resistance achieved with the TC-ion-CXL 6.75 J/cm^2^ protocol was closest to that of the standard epi-off-CXL 5.4 J/cm^2^ protocol, suggesting that this technique may be the best trans-epithelial alternative for epi-off-CXL. Its slightly lower efficacy compared to epi-off-CXL, demonstrated by measurements of corneal disk diameter and tissue dry weight, may be due to a reduced riboflavin stromal absorption or due to other factors. These might include absorption of UVA by riboflavin within the epithelium, resulting in shielding of the underlying stroma and a reduced UVA dosage to the stroma, although this is unlikely as a 3 min PBS wash of the ocular surface was performed before UVA exposure. Another possible cause of the reduced efficacy might be the oxygen consumption by the epithelium itself (Harvitt and Bonanno, 1998), which could reduce the amount of oxygen available to the stroma to drive the CXL process (McCall et al., 2010). Such problems may be overcome by additional manipulation of the UVA dosage, in terms of its intensity and duration and/or by increasing oxygen availability and require further investigation. One further explanation may be the limitation of our porcine model. As the porcine cornea has a much thicker corneal epithelium than the human cornea (90 μm and 50 μm respectively), the results of the current study should be regarded as a conservative assessment of the effectiveness of trans-epithelial CXL. Unfortunately, human donor corneas are unsuitable for examining the effects of CXL on enzymatic resistance due to the fact that donors are typically over 60 years old, and the naturally occurring cross-links which increase with age, may mask the effects of the CXL treatment under investigation. However, it would be interesting to repeat our methodology in the rabbit model which has a thinner epithelium (40 μm) and is closer in thickness to that of the human cornea. Such a study would complement our current findings by providing a more liberal estimate of the effectiveness of trans-epithelial cross-linking.

This study, which shows our St Thomas'/Cardiff modified iontophoresis protocol (TC-ion-CXL) to be more effective than other trans-epithelial CXL protocols at increasing the enzymatic resistance of the cornea supports the concept that iontophoresis-assisted CXL may, with modifications in terms of riboflavin concentration, duration of iontophoretic treatment, riboflavin soak-time and UVA energy dose, be an effective technique to prevent the progression of keratoconus and avoid the postoperative pain associated with epithelial debridement. Such a technique is envisaged to be especially useful for eyes that are not eligible for treatment with epi-off-CXL due to minimum corneal thickness values less than 400 μm. Further laboratory studies to optimize this protocol and randomized, prospective clinical studies to compare its efficacy with epi-off CXL are indicated and are currently being undertaken (O'Brart D, personal communication, International Standard Randomized Controlled Trials Number: 04451470).

## Financial disclosure

No author has a financial or proprietary interest in any material or method mentioned.

## Conflict of interest

The authors have no conflicts of interest to disclose.

## Figures and Tables

**Table 1:** Treatment groups.

Group	Abbreviation	Epithelium	Riboflavin formulation	Ionto (1 mA)	Ribo soak	Ionto (0.5 mA)	Ribo soak	Saline rinse	9 mW UVA	Applied during irradiation
(1)Epithelium-off non-irradiated control	Epi-off-ribo	Off	Mediocross D: 0.1% Riboflavin, 20% dextran	–	30 min	–	–	–	–	–
(2) Epithelium-off standard CXL	Epi-off-CXL 5.4 J/cm^2^	Off	Mediocross D	–	30 min	–	–	–	10 min	Mediocross D
(3) Disrupted epithelium non-irradiated control	Dis-ribo	Disrupted	Vitamin B2 Streuli: 0.1% riboflavin, saline	–	30 min	–	–	5 min	–	–
(4) Disrupted epithelium CXL	Dis-CXL 5.4 J/cm^2^	Disrupted	Vitamin B2 Streuli	–	30 min	–	–	5 min	10 min	PBS
(5) Epithelium intact non-irradiated control	Medio-ribo	On	Mediocross TE: 0.25% riboflavin, 1.2% HPMC, 0.01% BACS, Pi-water	–	30 min	–	–	5 min	–	–
(6) Epithelium intact high riboflavin concentration CXL	Medio-CXL 5.4 J/cm^2^	On	Mediocross TE	–	30 min	–	–	5 min	10 min	PBS
(7) Epithelium intact, high riboflavin concentration and prolonged iontophoresis non-irradiated control	TC-ion-ribo	On	Mediocross TE	5 min	5 min	5 min	5 min	5 min	–	–
(8) Epithelium intact, high riboflavin concentration and prolonged iontophoresis CXL	TC-ion-CXL 5.4 J/cm^2^	On	Mediocross TE	5 min	5 min	5 min	5 min	5 min	10 min	PBS
(9) Epithelium intact, high riboflavin concentration, prolonged iontophoresis and high UVA energy dose CXL	TC-ion-CXL 6.75 J/cm^2^	On	Mediocross TE	5 min	5 min	5 min	5 min	3 min	12 min 30 s	PBS
(10) Epithelium intact, basic iontophoresis protocol	Ion-CXL 5.4 J/cm^2^	On	Mediocross M: 0.1% riboflavin, 1.0% HPMC	5 min	–	–	–	3 min	10 min	PBS
(11) Epithelium intact, basic iontophoresis protocol with high UVA energy dose	Ion-CXL 6.75 J/cm^2^	On	Mediocross M	5 min	–	–	–	3 min	12 min 30 s	PBS

**Table 2:** Time taken for the complete tissue digestion to occur.

Groups	Time taken for complete digestion (in days)		
	Minimum	Maximum	Average (±SD)
**RUN 1**			
(1) Epi-off-ribo	11	12	11.5 ± 0.55
(2) Epi-off-CXL 5.4 J/cm^2^	39	40	39.5 ± 0.55
(3) Dis-ribo	11	13	11.8 ± 0.75
(4) Dis-CXL 5.4 J/cm^2^	15	16	15.6 ± 0.52
(5) Medio-ribo	11	12	11.6 ± 0.51
(6) Medio-CXL 5.4 J/cm^2^	14	15	14.6 ± 0.52
(7) TC-ion-ribo	11	12	11.3 ± 0.52
(8) TC-ion-CXL 5.4 J/cm^2^	32	33	32.2 ± 0.41
**RUN 2**			
(1) Epi-off-ribo	9	10	9.5 ± 0.55
(2) Epi-off-CXL 5.4 J/cm^2^	43	44	43.5 ± 0.55
(8) TC-ion-CXL 5.4 J/cm^2^	34	35	34.3 ± 0.52
(9) TC-ion-CXL 6.75 J/cm^2^	41	42	41.7 ± 0.52
(10) Ion-CXL 5.4 J/cm^2^	27	28	27.3 ± 0.52
(11) Ion-CXL 6.75 J/cm^2^	34	35	34.5 ± 0.55
